# Supplementary material for: Recurrence Patterns for Pancreatic Ductal Adenocarcinoma after Upfront Resection Versus Resection Following Neoadjuvant Therapy: A Comprehensive Meta-Analysis
Source: J Clin Med. 2020 Jul 6;9(7):2132. doi: 10.3390/jcm9072132 (PMC7408905; doi:10.3390/jcm9072132)
Supplement: Supplementary file 1 [file jcm-09-02132-s001.pdf]

Supplementary material

Table S1. Patient characteristics among all studies included within the meta-analysis.

Author	Age (years)*		Male (n)		Head of Pancreas Tumor (n)		Distant Metastases	
	NAT	US	NAT	US	NAT	US	NAT	US
Versteijne et al[26]	66 (59-71)	67 (60-73)	64	74	97	117	-	-
Lof et al[1]	63 ± 9.5[α]	65 ± 10.8 [α]	47	51	0	0	-	-
Yoshiya et al[2]	67.0±7.3	68.8±10.6	9	4	0	0	0	0
Groot et al[3]	62.7±9.4	65.8±10.5	128	501	-	-	-	-
Nagakawa et al[4]	-	-	136	161	245	245	-	-
Nurmi et al[5]	65 (40,83)	66 (45,82)	33	67	-	-	-	-
Jang et al[6]	59.4±8.4	58.9±11.3	17	15	23	17	0	0
Chen et al[7]	-	-	52	52	70	70	8	7
Masui et al[8]	63 (43,73)	66 (56,80)	8	6	13	13	0	0
Ferrone et al[9]	-	-	21	45	-	-	-	-
Fujii et al[10]	66 (45,76)	63 (42,82)	10	41	-	-	-	-
Ishikawa et al[11]	-	-	-	-	-	-	0	0
Golcher et al[12]	62.5 (33,76)	65.1 (46,73)	17	18	33	33	2	0
Papavasiliou et al[13]	66 (38,84)	67 (35,91)	64	83	108	201	-	-
Cho et al[14]	59.57±8.57	60.76±10.79	16	10	26	18	0	0
Jiang et al[15]	45.9±9.8	45.5±9.3	75	69	98	96	-	-
Kang et al[16]	59.3±9.1	62.1±9.1	14	63	12	48	-	-
Tajima et al[17]	62.6 (51,77)[β]	66.0 (52,80)[β]	7	14	9	11	-	-
Barugola et al[18]	59 (50,68)	64 (56,70)	21	202	39	291	0	0
Katz et al[19]	64.8 (34.5,85.4)	65.1 (24.9,84.5)	79	24	147	47	-	-
Barbier et al[20]	65 (39,81)	64 (37,79)	-	-	38	67	-	-
Sahora et al[21]	-	-	17	-	26	-	0	-
Greer et al[22]			-	-	-	-	-	-
Massucco et al[23]	62.7±5.6	66.5±8.7	16	20	23	41	-	-
Moutardier et al[24]	65 (39,76)	65 (50,80)	24	9	39	17	0	0
Pingpank et al[25]	68 (41,80)	67 (41,85)	18	21	53	47	-	-

The baseline characteristics are reported for all patients undergoing NAT irrespective of subsequent resection. PDAC Pancreatic duct adenocarcinoma; NAT Neoadjuvant therapy; US Upfront surgery; * Expressed as Mean ± Standard deviation or Median (Range); β Mean (Range); α Median and interquartile range; - Not reported.

Table S2. Pathological outcomes in all studies within the meta-analysis.

Author	Poor Tumor Differentiation		N0 Nodal Status (n)		Tumour Size (mm)*		Perineural invasion (n)		Lymphovasc-ular invasion (n)		R0 resection (n)	
	NAT	US	NAT	US	NAT	US	NAT	US	NAT	US	NAT	US
Versteijne et al[26]	-	-	48	20	-	-	28	67	14	33	51	37
Lof et al[1]	-	-	45	31	-	-	70	80	45	66	59	64
Yoshiya et al[2]	-	-	8	6	41.3±20.0	42.7±18.6	-	-	5[±]	8[±]	9	6
Groot et al[3]	69	366	91	719	25±15	32±15	133	859	91	544	180	658
Nagakawa et al[4]	-	-	126	220	-	-	-	-	133	84	191	195
Nurmi et al[5]	14	26	36	108	25 (20,30)[¥]	30 (25,40)[¥]	47	120	16	58	58	106
Jang et al[6]	-	-	10	15	34±08	35±9	-	-	27	23	14	6
Chen et al[7]	41	42	44	45	-	-	-	-	-	-	-	-
Masui et al[8]	5	5	10	14	33 (18,50)	32 (17,75)	2	4	9	7	12	10
Ferrone et al[9]	-	-	14	69	25 (1,55)	32 (15,107)	29	83	14	61	35	75
Fujii et al[10]	5	9	3	46	29 (12,40)	30 (20,60)	3	24	8	36	0	30

Ishikawa et al[11]	-	-	17	167	-	-	-	-	-	-	-	-
Golcher et al[12]	-	-	11	3	-	-	-	-	-	-	17	16
Papavasiliou et al[13]	30	70	36	158	25 (0,105)	30 (10,85)	44	121	-	-	74	126
Cho et al[14]	-	-	9	9	16.5±9.2	26.9±6.4	-	-	13	8	29	20
Jiang et al[15]	23	25	-	-	32±13	35±08	-	-	-	-	95	96
Kang et al[16]	3	7	10	55	-	-	10	46	4	20	28	92
Tajima et al[17]	-	-	10	12	30.1 (16,55)$^{\beta}$	30.6 (17,53)$^{\beta}$	13	19	11	19	11	18
Barugola et al[18]	3	115	13	312	20 (15,30)$^{¥}$	25 (20,30)$^{¥}$	-	-	-	-	29	216
Katz et al[19]	27	6	75	41	24 (3,80)	30 (13,55)					138	41
Barbier et al[20]												
Sahora et al[21]	-	-	-	-	15 (0,80)	30 (8,80)	10	56	4	47	35	45
Greer et al[22]	3	-	9	-	-	-	-	-	-	-	9	-
Massucco et al[23]	-	-	-	-	-	-	-	-	-	-	31	-
Moutardier et al[24]	3	18	2	36	-	-	3	25	-	-	7	30
Pingpank et al[25]	5	-	3	11	32 (12,44)	31 (15,55)	-	-	-	-	21	11
Yoshiya et al[2]	-	-	-	-	25 (20,55)	40 (15,50)	-	-	-	-	27	11

Pathological outcomes are provided for patients undergoing resection. NAT Neoadjuvant therapy; US Upfront surgery; * Expressed as Mean ± Standard deviation or Median (Range); β Mean (Range); ¥ Median (Inter-quartile range); ± Included only vascular invasion; - Not reported.

Table S3. Resectability and Neoadjuvant therapy of all patients who underwent resection included in the quantitative analysis within this meta-analysis.

Author	Preoperative Tumor Resectability (n)		Type of NAT (n)*						Preoperative Radiotherapy (n)
	NAT	US	Gem-citabine	Plant Alkaloids	Platinum based	Folfirinox	Fluoropyrimidines	Comb-ination	
**Versteijne et al[26]**	-	-	72	-	-	-	**-**	0	72
Lof et al[1]	-	-	-	-	-	-	**-**	-	-
Yoshiya et al[2]	BR (8) UR (3)	BR (9)	**11**$^{\alpha}$	7$^{\pm}$	-	-	3$^{\pm}$	10	-
Groot et al[3]	BR (138) LA (93)	-	**133**$^{€}$	-	-	**142**$^{€}$	-	44	180
Nagakawa et al[4]	BR (223)	BR (277)	-	-	-	-	-	-	-
Nurmi et al[5]	BR (75)	BR (150)	**75**$^{\beta}$	-	-	-	-	75	-
Jang et al[6]	BR (17)	BR (18)	17	-	-	-	-	0	17
Chen et al[7]	LA (98)	La (98)	-	-	-	-	-	-	-

Masui et al[8]	BR (15)	BR (19)	15¥	-	-	-	**15¥**	15	-
Ferrone et al[9]	BR (9)<br>LA (19)<br>R (12)	R (87)	-	-	-	40	-	0	-
Fujii et al[10]	BR (18)	BR (50)	-	-	-	-	18	0	33
Ishikawa et al[11]	-	-	112	-	-	-	-	0	112
Golcher et al[12]	R (33)	R (33)	**33¥**	-	-	-	33¥	33	-
Papavasiliou et al[13]	-	-	85	-	-	-	23	0	108
Cho et al[14]	BR (30)	BR (21)	30	-	-	-	-	0	30
Jiang et al[15]	-	-	**43Ω**	-	8±	-	38	8	0
Kang et al[16]	BR (32)	R (104)	32	-	-	-	-	0	32
Tajima et al[17]	R (13)	R (21)	13¥	-	-	-	**13¥**	13	-
Barugola et al[18]	BR (27)<br>LA (14)	R (362)	**41£**	-	10±	-	9±	19	24
Katz et al[19]	BR (41)<br>R (106)	LA (1)<br>R (46)	81	-	-	-	64	0	145
Barbier et al[20]	-	-	-	-	38¥	-	**38¥**	38	38
Sahora et al[21]	-	-	**13¥**	-	13¥	-	-	13	-
Greer et al[22]	BR (20)<br>UR (6)<br>R (16)	BR (8)<br>UR (1)<br>R (32)	**37**	16±	5¥	-	5¥	21	42
Massucco et al[23]	BR (7)<br>UR (1)	R (44)	8	-	-	-	-	0	8
Moutardier et al[24]	R (23)	R (17)	-	-	23¥	-	**23¥**	23	23
Pingpank et al[25]	-	-	-	-	-	-	-	-	24

Pathological Resectability and Neoadjuvant therapy are provided for patients undergoing resection. NAT Neoadjuvant therapy; US Upfront surgery; BR Borderline Resectable; Locally Advanced; UR Unresectable; R Resectable; FU Fluorouracil based therapy (including S-1 and 5-FU); * The total number receiving this NAT including combination therapies so this will not add up to the total NAT cohort population as combination therapy is entered into multiple NAT columns; ¥ In combination with each other in the corresponding row; ± In combination with Gemcitabine; € n=44 were in combination with Gemcitabine and Folfirinox; β in combination with an unreported agent; α Gemcitabine alone in 1 patient; £ Gemcitabine alone in 22 patients; Ω Gemcitabine alone in 35 patients; - Not reported; **BOLD** indicates the base NAT if in combination.

Table S4. Results of the mixed-effects meta-regression to identify potential predictors of overall recurrence in the A) neoadjuvant therapy (NAT) and B) up-front surgery (US) groups. Variables were included in the mixed effects model if they were significantly different between NAT and US, where differences were identified on meta-analysis and with an incidence of ≥ three for categorical variables.

Variable	Estimate	Standard Error	P Value	95% Confidence Interval
Borderline Resectable tumor status	0.1190	0.0305	0.030	0.022, 0.216
R0 resection	-0.258	0.096	0.074	-0.562, 0.046
N0 nodal status	-0.729	0.084	0.003	-0.996, -0.461
Perineural Invasion	0.002	0.0004	0.008	0.001, 0.003

A) NAT.

Variable	Estimate	Standard Error	P Value	95% Confidence Interval
Borderline Resectable tumor status	0.075	0.050	0.166	-0.038, 0.188
Resectable tumor status	-0.210	0.050	0.002	-0.323, -0.098
R0 resection	0.098	0.067	0.178	-0.054, 0.251
N0 nodal status	-0.047	0.059	0.448	-0.181, 0.087
Perineural Invasion	0.045	0.052	0.416	-0.074, 0.163

B) US.

Table S5. A) The MINORS criteria[27] scores for all non-randomsied studies included in the review and B) Cochranes Risk of bias tool for randomised trials.

Author	Item /2												Total
	1	2	3	4	5	6	7	8	9	10	11	12	/24
**Lof et al[1]**	2	2	0	2	0	2	0	0	2	2	1	2	15
Yoshiya et al[2]	2	1	0	2	0	2	0	0	1	2	0	1	11
Groot et al[3]	2	1	0	2	0	1	1	0	1	0	0	2	10
Nagakawa et al[4]	1	2	2	2	0	2	0	0	2	2	1	2	16
Nurmi et al[5]	2	1	0	2	0	2	2	0	2	1	1	2	15
Chen et al[7]	1	1	0	2	0	2	0	0	2	1	1	1	11
Masui et al[8]	2	2	2	2	0	2	1	0	2	2	2	2	19
Ferrone et al[9]	2	2	0	2	0	1	0	0	1	2	0	2	12
Fujii et al[10]	2	1	0	2	0	1	0	0	1	2	0	2	11
Ishikawa et al[11]	2	1	0	1	0	2	0	0	2	2	0	2	12
Papavasiliou et al[13]	1	2	0	2	0	2	0	0	2	2	0	2	13
Cho et al[14]	2	2	0	2	0	2	0	0	2	2	1	2	15
Jiang et al[15]	1	2	0	2	0	2	0	0	1	2	2	2	14
Kang et al[16]	1	2	0	1	0	2	0	0	2	2	0	2	12
Tajima et al[17]	1	2	0	1	0	1	0	0	1	2	1	2	11
Barugola et al[18]	2	2	0	2	0	1	0	0	2	2	1	2	14
Katz et al[19]	2	1	0	2	0	2	0	0	2	2	0	2	13
Barbier et al[20]	1	2	0	2	0	1	0	0	1	2	1	2	12
Sahora et al[21]	1	2	2	2	0	1	1	0	2	2	0	2	15
Greer et al[22]	1	2	0	2	0	2	0	0	2	2	0	2	13
Massucco et al[23]	1	2	0	2	0	2	0	0	2	2	0	2	13
Moutardier et al[24]	1	2	0	2	0	1	0	0	2	2	1	2	13
Pingpank et al[25]	2	1	0	2	0	2	0	0	2	2	0	2	13

A)MINORS.

Author	Random Sequence Generation	Allocation concealment	Selective reporting	Blinding (Participants and Personnel)	Blinding (Outcome)	Incomplete outcome data	Other sources of bias
Versteijne et al[26]	Low	U	Low	High	High	Low	U
Golcher et al[12]	U	U	Low	High	High	Low	Low
Jang et al[6]	Low	U	Low	High	High	Low	U

B) Cochranes Risk of bias tool *Item 1* Clear aim, *Item 2* consecutive patients, *Item 3* prospective data collection, *Item 4* appropriate end points, *Item 5* unbiased assessment, *Item 6* appropriate follow-up, *Item 7* insignificant lost to follow-up, *Item 8* power calculation, *Item 9* appropriate control, *Item 10* Contemporary cohorts, *Item 11* Equivalent baseline characteristics, *Item 12* appropriate statistical analysis. *High* High risk of bias, *Low* Low risk of bias, *U* Unclear risk of bias. [2-25].

A) Locoregional recurrence

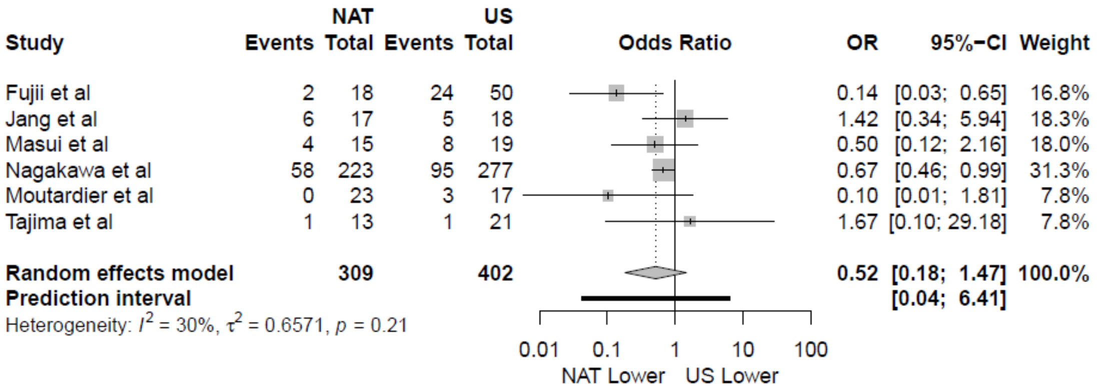

B) Distant recurrence

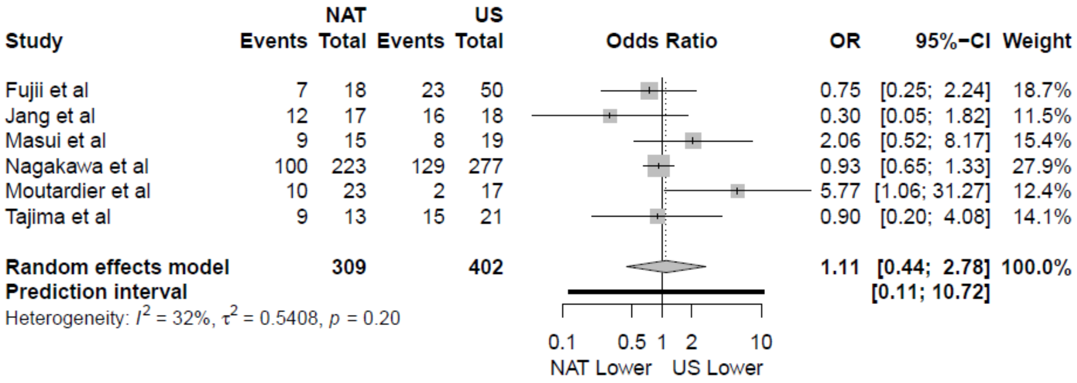

Figure S1. Forest plot showing rate of locoregional (A) and distant (B) recurrence in studies reporting solely either borderline resectable (BRPC) or resectable (RPC) pancreatic cancer for Neoadjuvant therapy (NAT) vs Up-front Surgery (US). A Manel–Haenszel random effects model with a Hartung–Knapp adjustment was used for the meta-analysis of all outcomes. A Sidik–Jonkman estimator was utilised for tau^2. Odds ratios (OR) are shown with 95 percent confidence intervals (CI).

A) Two-Year Recurrence-Free Survival

Study	NAT Events	NAT Total	US Events	US Total	OR	95%−CI	Weight
Lof et al	23	94	21	94	1.13	[0.57; 2.21]	9.4%
Barugola et al	12	41	128	362	0.76	[0.37; 1.53]	9.2%
Cho et al	14	30	5	21	2.80	[0.82; 9.62]	5.8%
Fujii et al	10	18	8	50	6.56	[1.98; 21.75]	5.9%
Golcher et al	8	33	7	33	1.19	[0.38; 3.77]	6.2%
Greer et al	39	42	30	41	4.77	[1.22; 18.62]	5.1%
Jiang et al	50	112	34	120	2.04	[1.18; 3.52]	10.4%
Katz et al	65	147	13	47	2.07	[1.01; 4.25]	9.1%
Massucco et al	2	8	8	44	1.50	[0.25; 8.84]	3.6%
Masui et al	4	15	4	19	1.36	[0.28; 6.68]	4.2%
Papavasiliou et al	28	106	36	166	1.30	[0.73; 2.29]	10.2%
Sahora et al	3	13	4	73	5.17	[1.01; 26.61]	4.0%
Tajima et al	4	13	8	21	0.72	[0.17; 3.14]	4.7%
Versteijne et al	34	91	30	104	1.47	[0.81; 2.68]	10.0%
Yoshiya et al	6	11	1	9	9.60	[0.88; 105.17]	2.3%
**Random effects model**		**774**		**1204**	**1.79**	**[1.23; 2.61]**	**100.0%**
**Prediction interval**						**[0.50; 6.44]**	

Heterogeneity: $I^2 = 34\%$, $\tau^2 = 0.3198$, $p = 0.09$

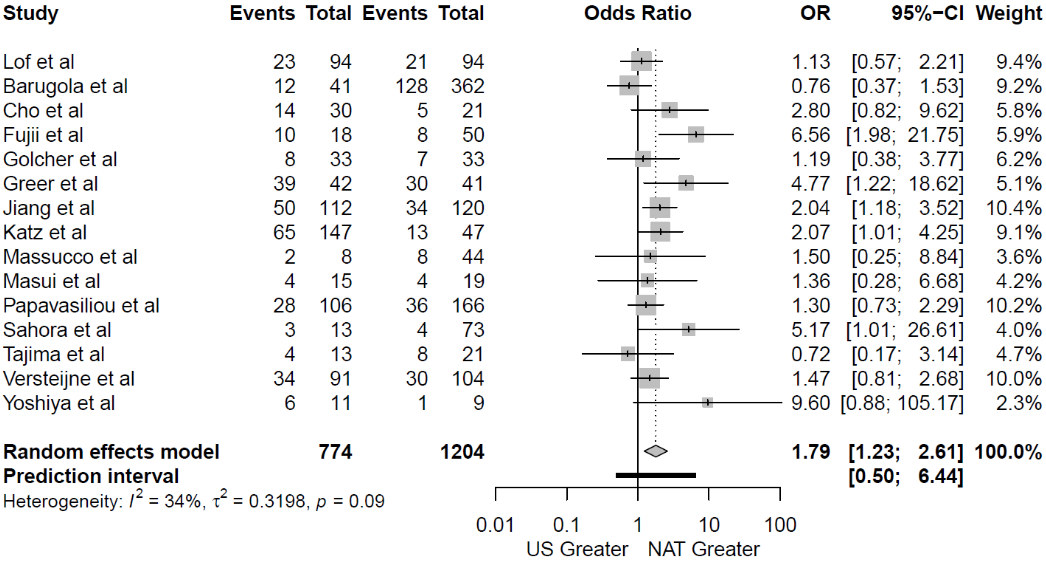

B) Five-Year Recurrence-Free Survival

Study	NAT Events	NAT Total	US Events	US Total	OR	95%−CI	Weight
Barugola et al	9	41	80	362	0.99	[0.45; 2.16]	14.8%
Chen et al	16	98	12	98	1.40	[0.62; 3.13]	14.6%
Cho et al	13	30	3	21	4.59	[1.11; 18.98]	9.4%
Golcher et al	1	33	2	33	0.48	[0.04; 5.62]	4.6%
Greer et al	39	42	22	41	11.23	[2.98; 42.24]	10.1%
Jiang et al	15	112	9	120	1.91	[0.80; 4.55]	14.0%
Katz et al	43	147	6	47	2.83	[1.12; 7.14]	13.4%
Masui et al	2	15	2	19	1.31	[0.16; 10.56]	5.9%
Papavasiliou et al	8	106	11	166	1.15	[0.45; 2.96]	13.3%
**Random effects model**		**624**		**907**	**1.94**	**[1.03; 3.69]**	**100.0%**
**Prediction interval**						**[0.33; 11.36]**	

Heterogeneity: $I^2 = 45\%$, $\tau^2 = 0.4799$, $p = 0.07$

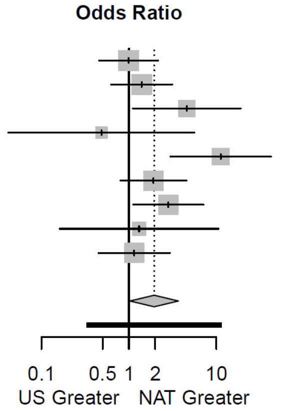

Figure S2. Forest plot showing recurrence-free survival at A) 2-years and B) 5-years postoperatively following Neoadjuvant therapy (NAT) vs Up-front Surgery (US). A Manel–Haenszel random effects model with a Hartung–Knapp adjustment was used for the meta-analysis of all outcomes. A Sidik–Jonkman estimator was utilised for tau². Odds ratios (OR) are shown with 95 percent confidence intervals (CI).

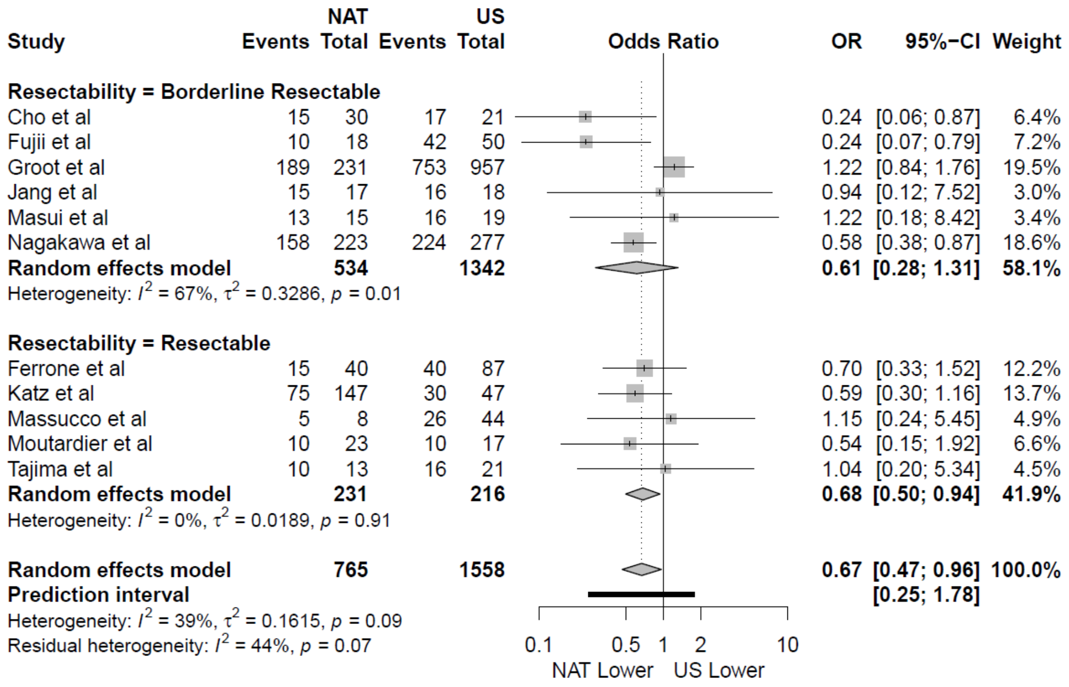

A)Resectability

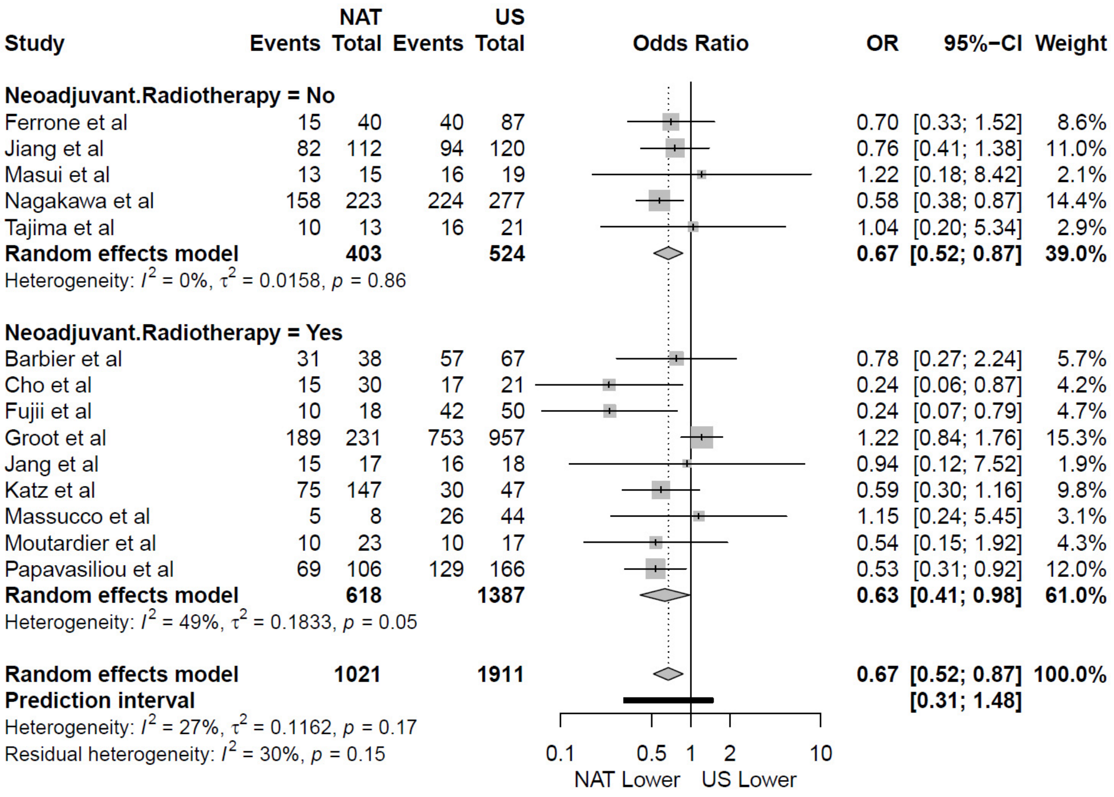

B) Preoperative chemoradiotherapy

Figure S3. Forest plot showing overall recurrence rates in articles reporting A) preoperative resectability and B) preoperative chemoradiotherapy subgroups following Neoadjuvant therapy (NAT) vs Up-front Surgery (US). A Manel–Haenszel random effects model with a Hartung– Knapp adjustment was used

for the meta-analysis of all outcomes. A Sidik–Jonkman estimator was utilised for tau². Odds ratios (OR) are shown with 95 percent confidence intervals (CI).

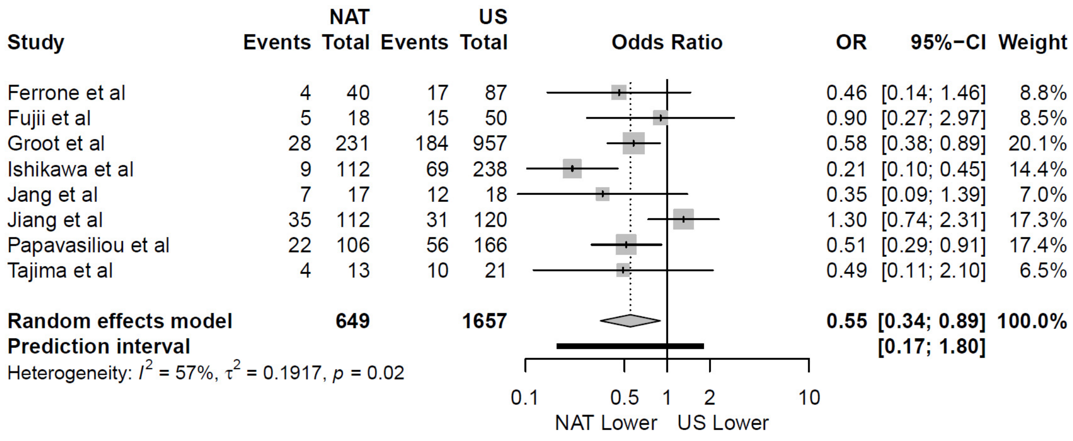

A) Liver Recurrence.

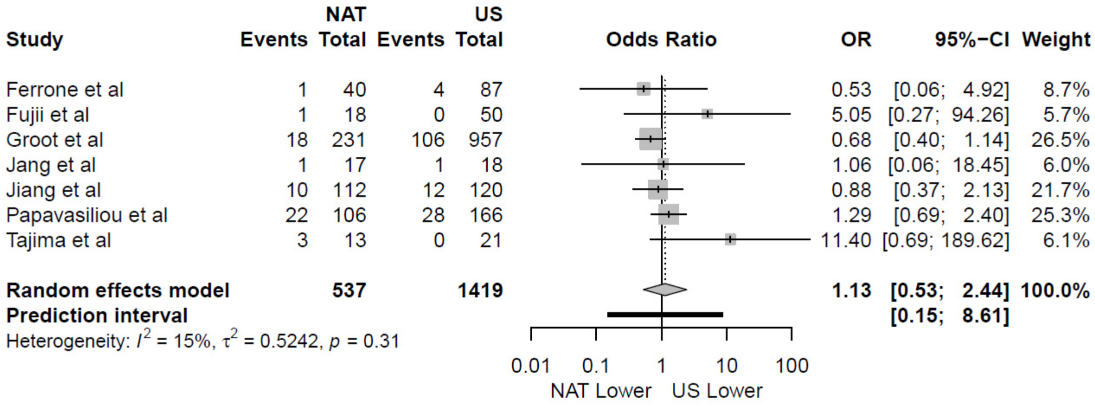

B) Lung Recurrence.

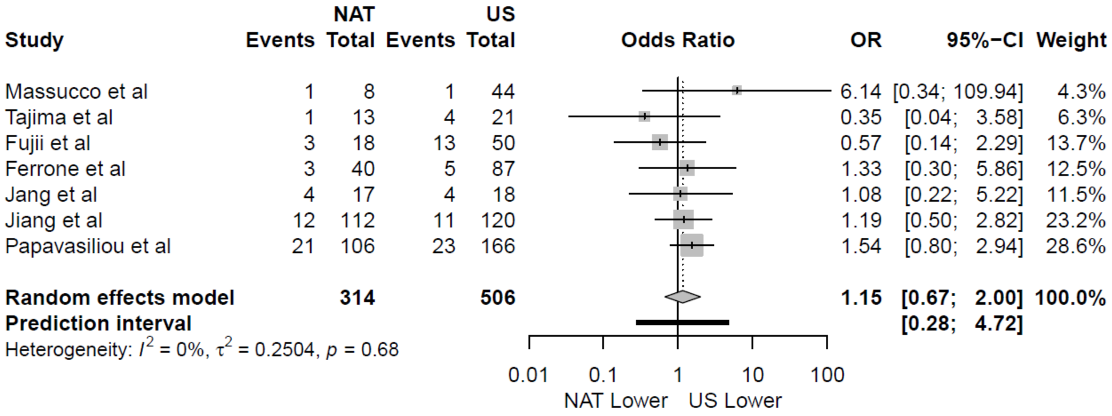

C) Peritoneal Recurrence.

Figure S4. Forest plot showing A) Liver, B) Lung and B) Peritoneal recurrence rates following Neoadjuvant therapy (NAT) vs Up-front Surgery (US). A Manel–Haenszel random effects model with

a Hartung–Knapp adjustment was used for the meta-analysis of all outcomes. A Sidik–Jonkman estimator was utilised for tau². Odds ratios (OR) are shown with 95 percent confidence intervals (CI).

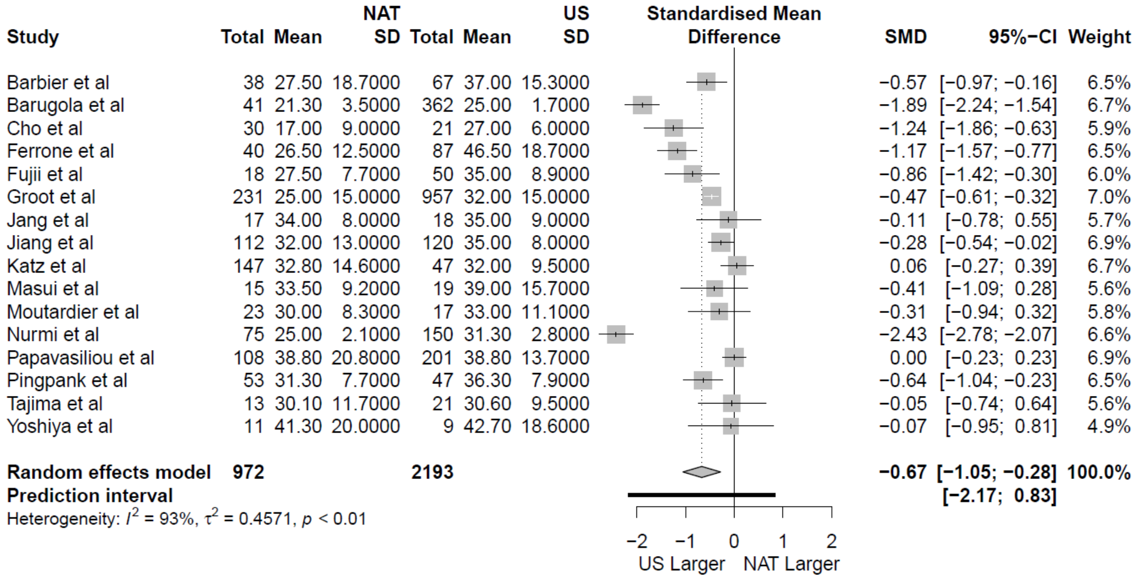

Figure S5. Forest plot showing mean tumor diameter in mm following Neoadjuvant therapy (NAT) vs Up-front Surgery (US). A Manel–Haenszel random effects model with a Hartung–Knapp adjustment was used for the meta-analysis of all outcomes. A Sidik–Jonkman estimator was utilised for tau². Standard Mean Differences (SMD) are shown with 95 percent confidence intervals.

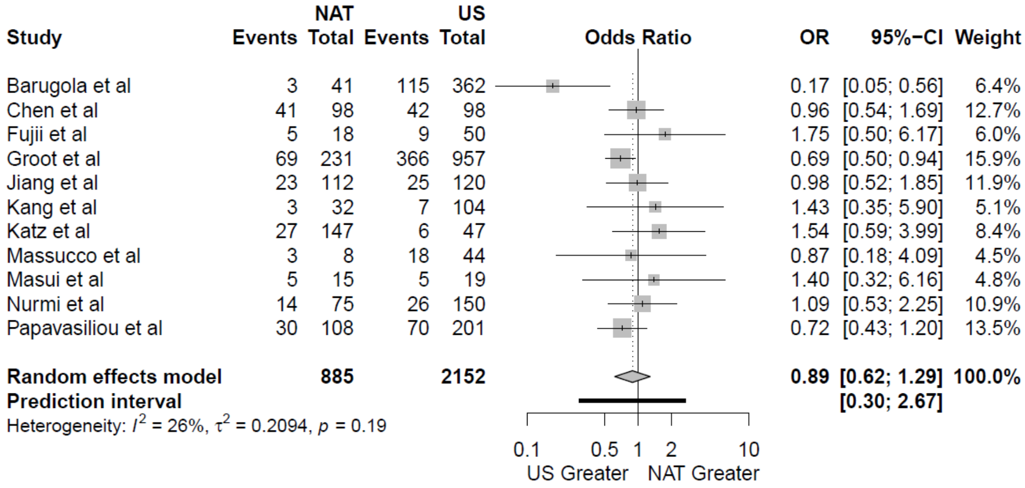

A) Poor Tumor Differentiation.

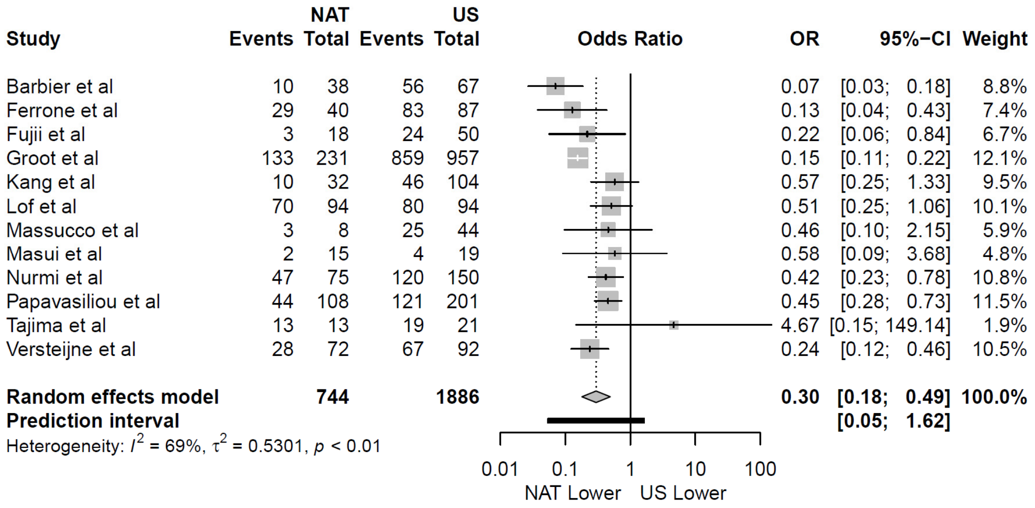

B) Perineural Involvement.

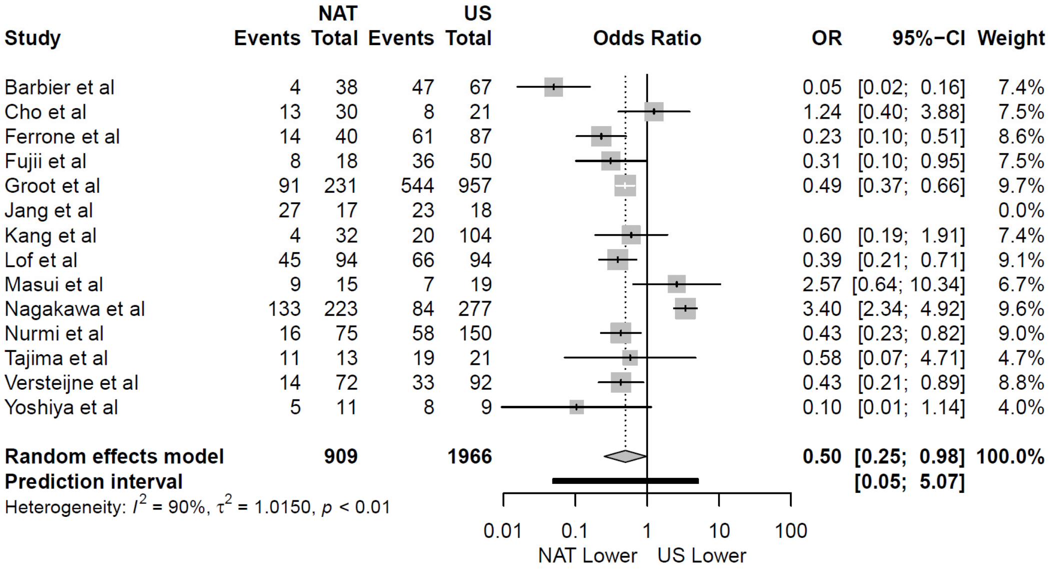

C) Lymphovascular Involvement.

Figure S6. Forest plot showing A) Poor Tumor differentiation at the time of surgery B) perineural and A) Lymphovascular involvement rates following Neoadjuvant therapy (NAT) vs Up-front Surgery (US). A Manel–Haenszel random effects model with a Hartung-Knapp adjustment was used for the meta-analysis of all outcomes. A Sidik–Jonkman estimator was utilised for tau². Odds ratios (OR) are shown with 95 percent confidence intervals (CI).

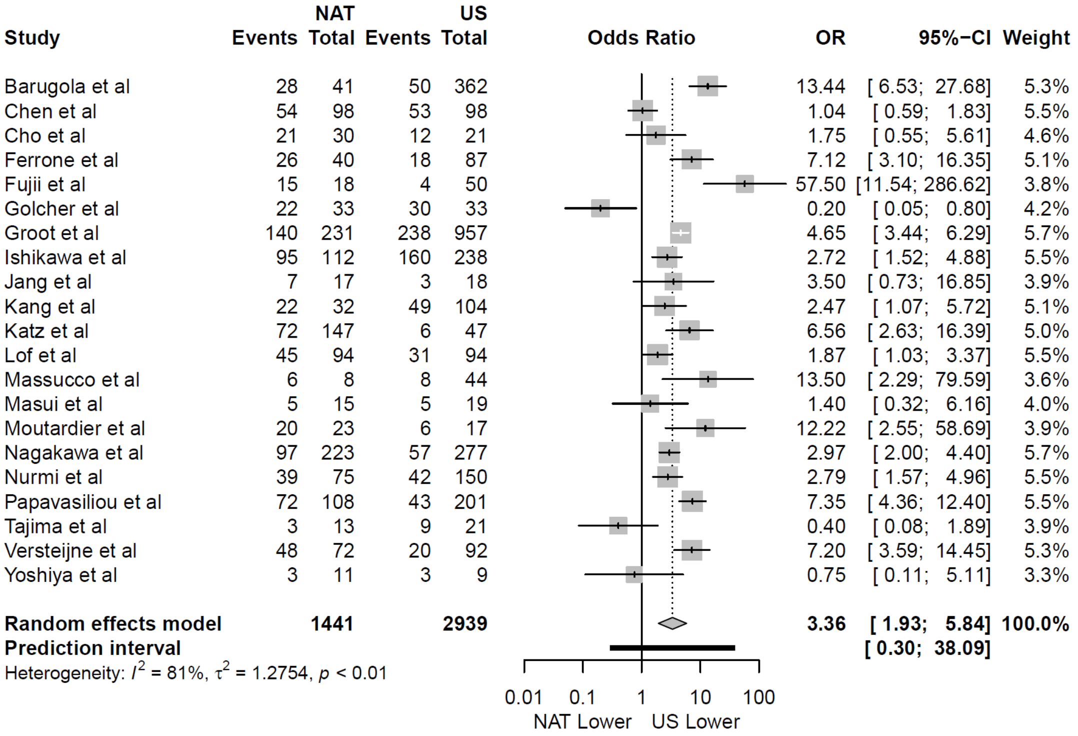

A) N0 Nodal Status.

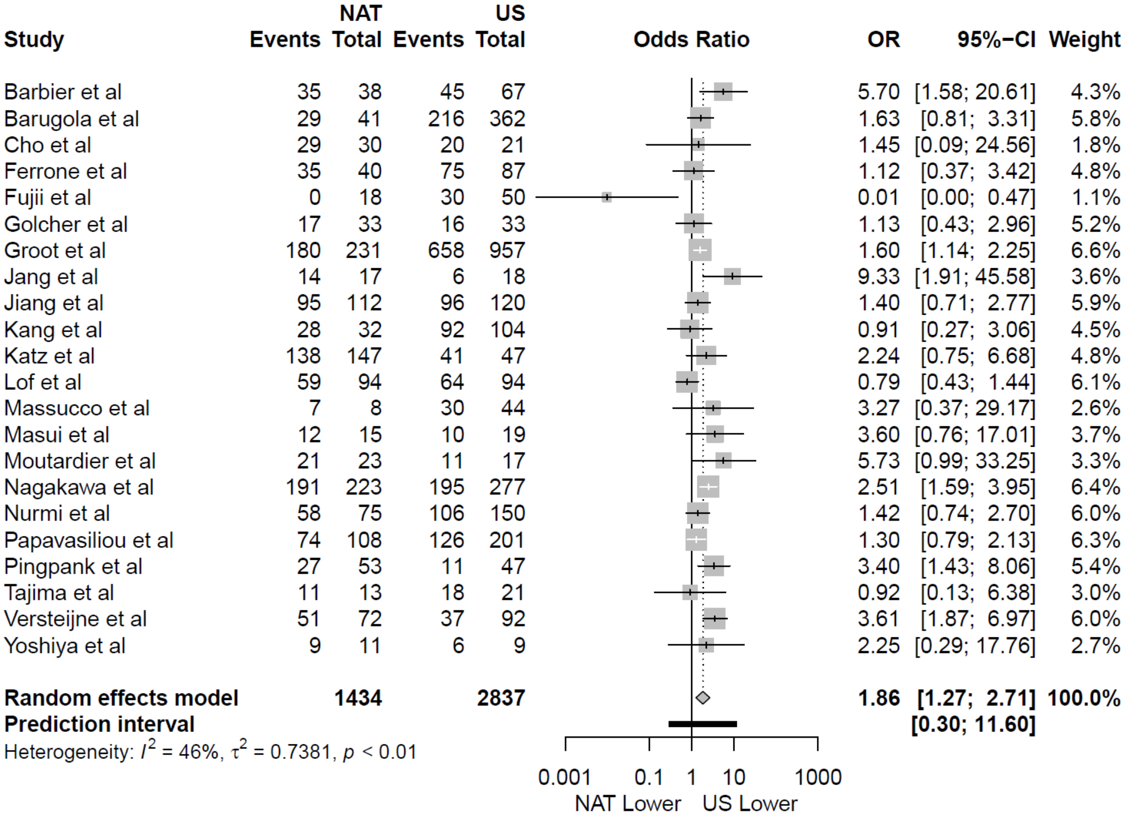

B) R0 Resection.

Figure S7. Forest plot showing A) N0 Status and B) R0 Resection rates following Neoadjuvant therapy (NAT) vs Up-front Surgery (US). A Manel–Haenszel random effects model with a Hartung–Knapp adjustment was used for the meta-analysis of all outcomes. A Sidik–Jonkman estimator was utilised for tau^2. Odds ratios (OR) are shown with 95 percent confidence intervals (CI).

Tumor diameter

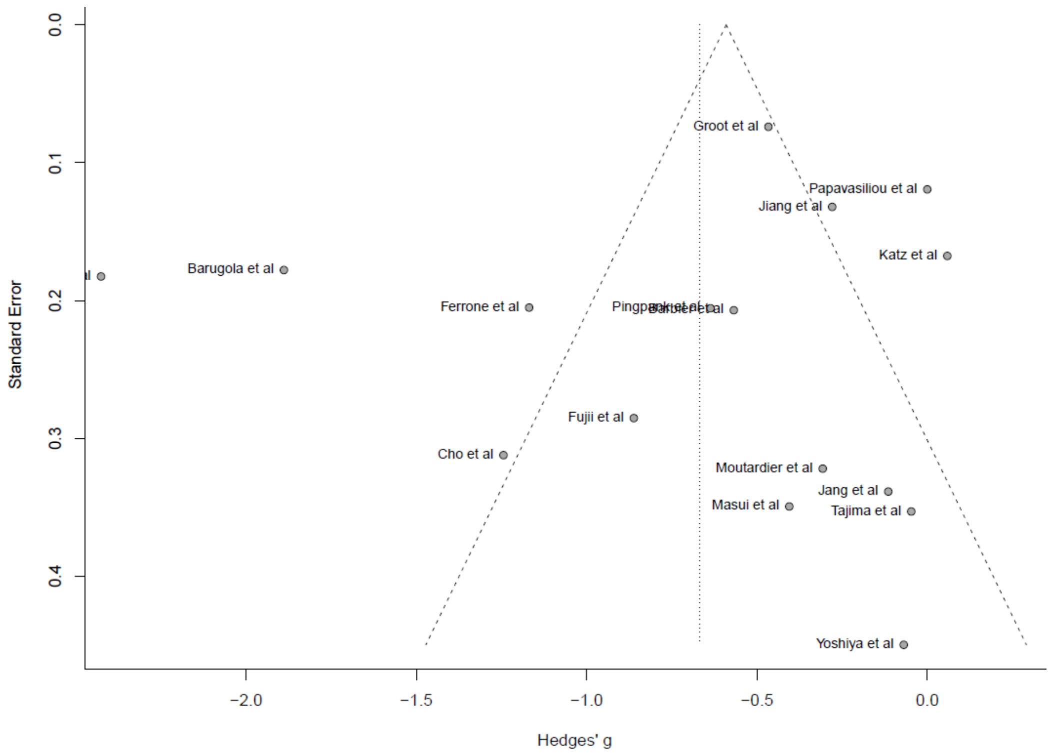

A) N0 nodal status

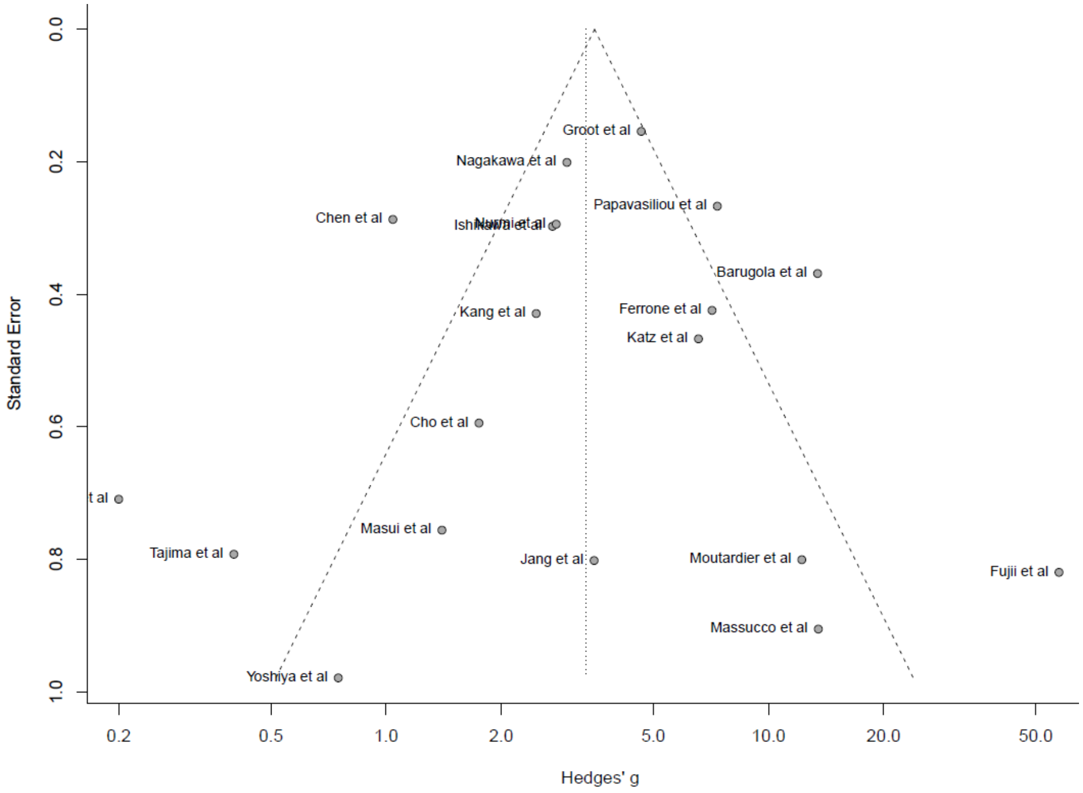

B) Poor tumor differentiation

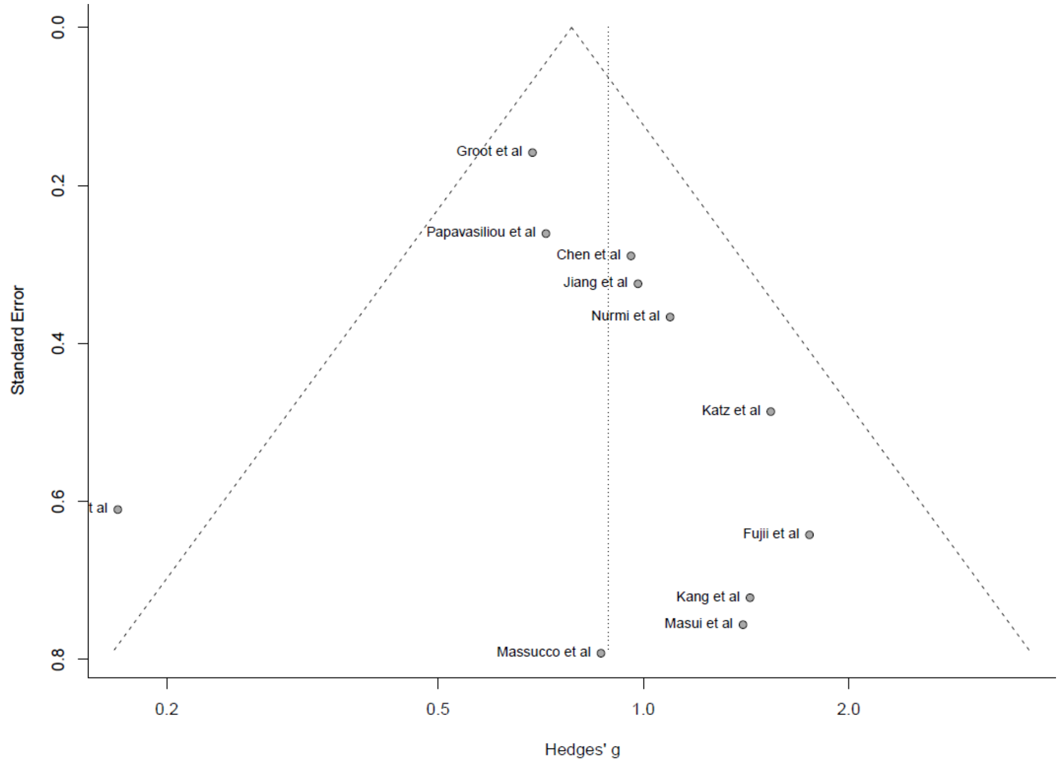

C) Perineural invasion

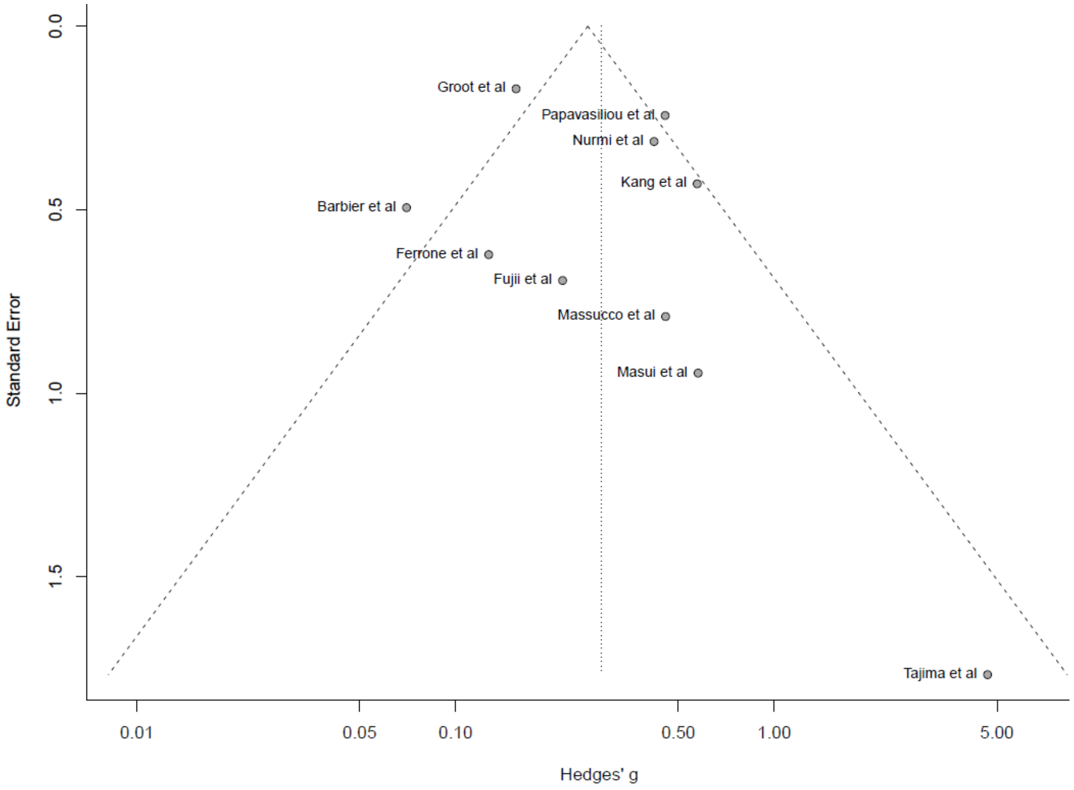

D) Lymphovascular invasion

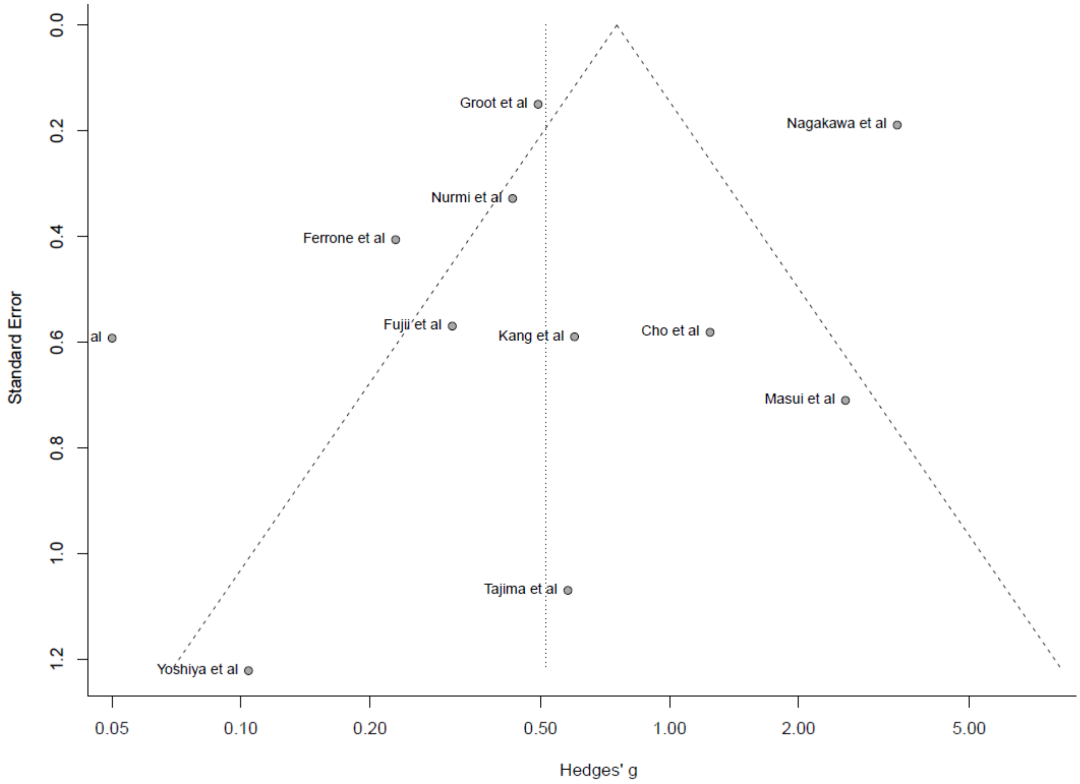

E) R0 Resection

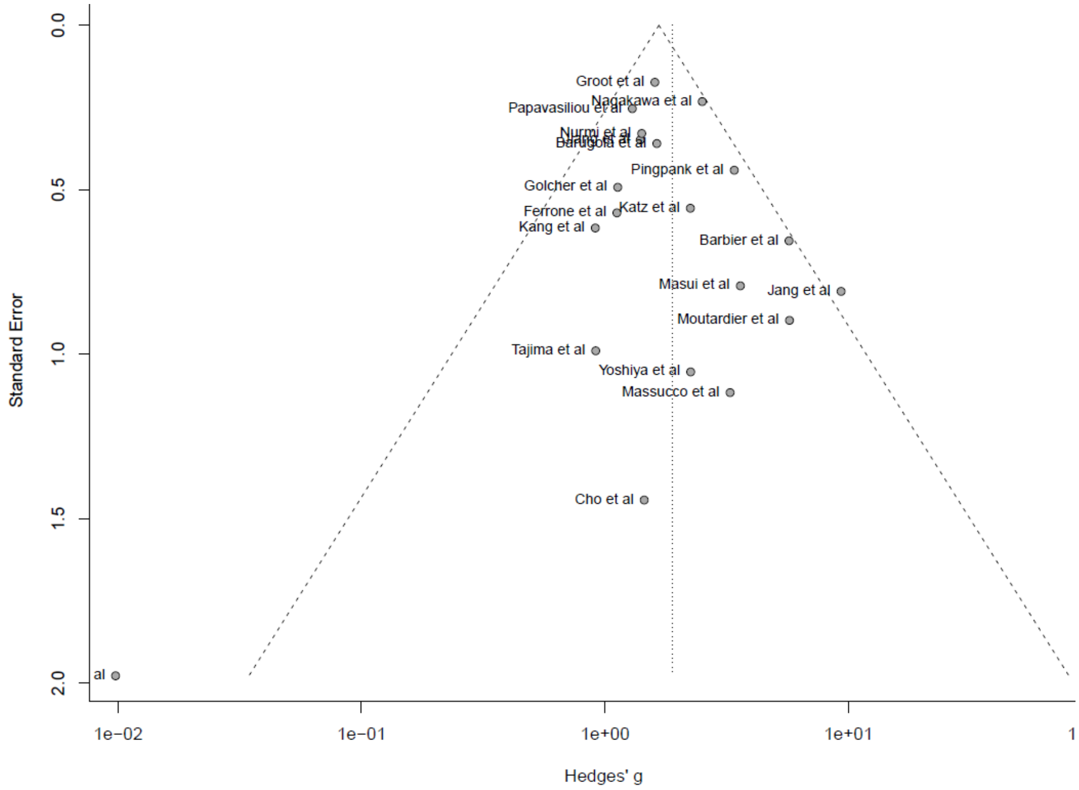

F) Overall Recurrence

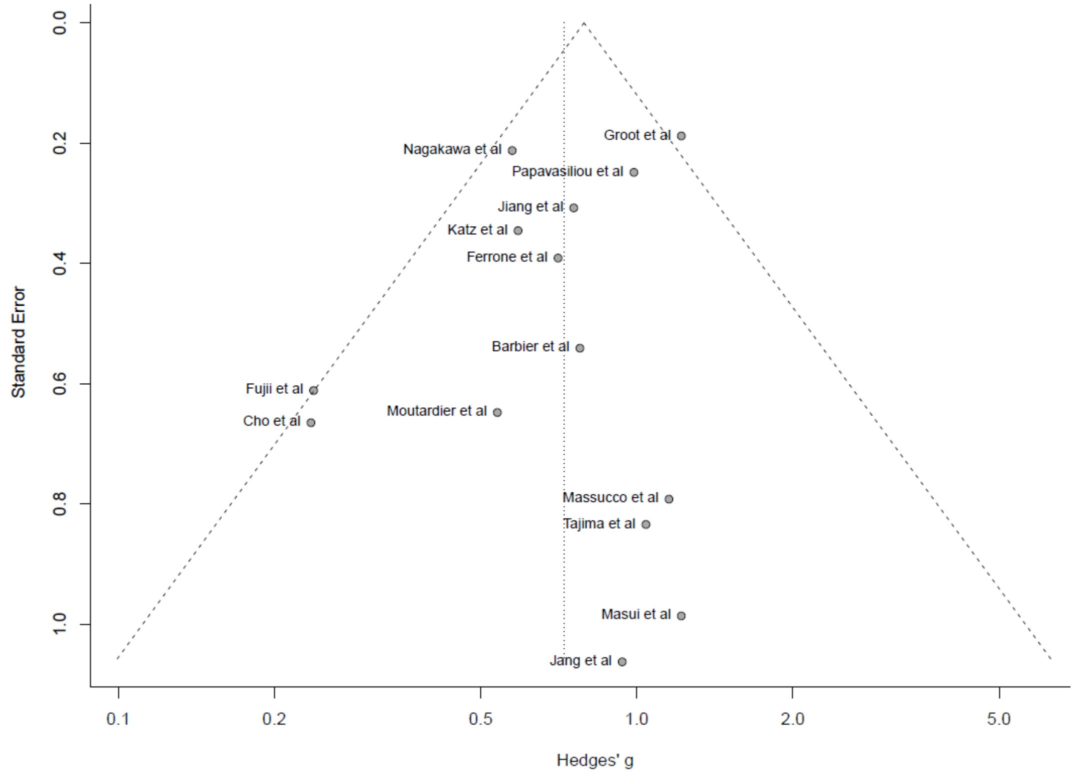

G) Time to first recurrence

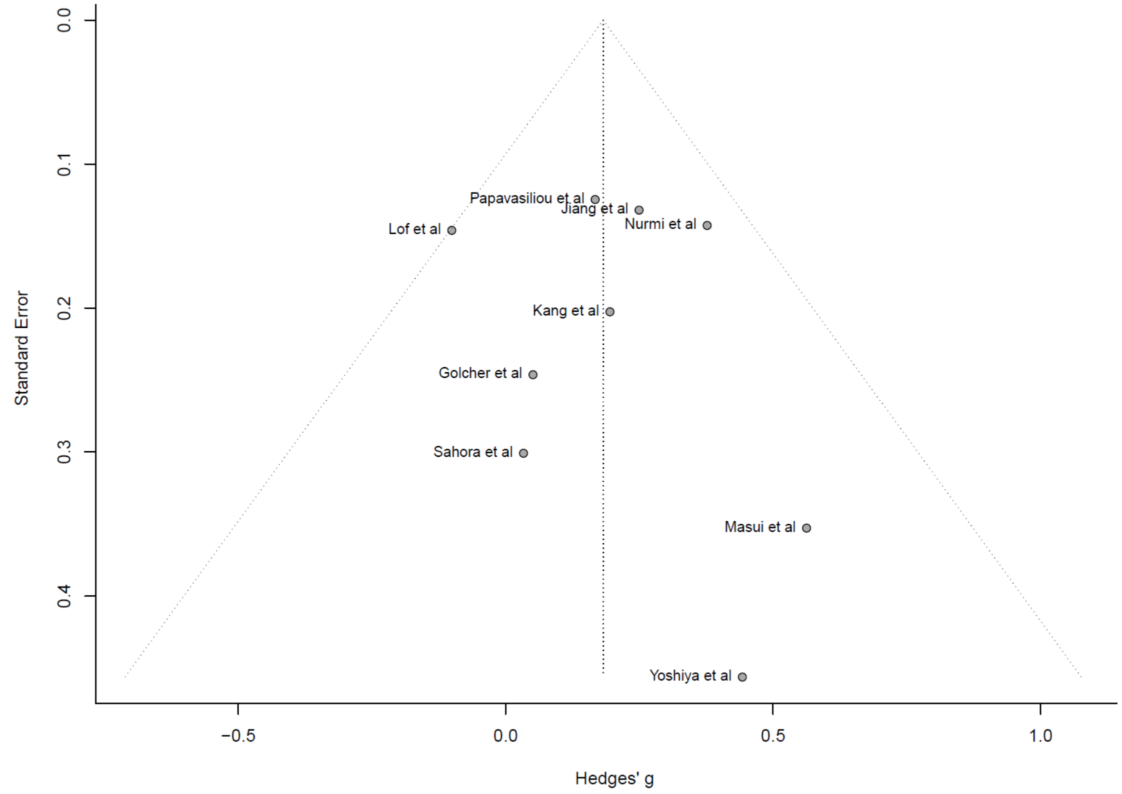

Figure S8. Funnel plots for all meta-analysis outcomes including A) Tumor Size, B) N0 Nodal Status, C) Poor tumor differentiation, D) Perineural invasion, E) Lymphovascular invasion, F) R0 Resection, G) Overall recurrence, and H) Recurrence-free survival.
